# Chronological transitions of hepatocyte growth factor treatment effects in spinal cord injury tissue

**DOI:** 10.1186/s41232-024-00322-9

**Published:** 2024-03-13

**Authors:** Yuji Okano, Yoshitaka Kase, Yu Suematsu, Masaya Nakamura, Hideyuki Okano

**Affiliations:** 1 Department of Physiology, Keio University School of Medicine, 35, Shinanomachi, Shinjuku-Ku, Tokyo, 160-8582 Japan; 2 Department of Extended Intelligence for Medicine, The Ishii-Ishibashi Laboratory, Keio University School of Medicine, 35, Shinanomachi, Shinjuku-Ku, Tokyo, 160-8582 Japan; 3 Division of CNS Regeneration and Drug Discovery, International Center for Brain Science (ICBS), Fujita Health University, 1-98 Dengakugakubo, Kutsukake-Cho, Toyoake-Shi, Aichi 470-1192 Japan; 4 Department of Orthopaedic Surgery, Keio University School of Medicine, 35 Shinanomachi, Shinjuku-Ku, Tokyo, 160-8582 Japan

**Keywords:** RNA-seq data analysis, Spinal cord injury, Hepatocyte growth factor, Neuronal development, Inflammatory response

## Abstract

Inflammatory responses are known to suppress neural regeneration in patients receiving stem cell-based regenerative therapy for spinal cord injury (SCI). Consequently, pathways involved in neurogenesis and immunomodulation, such as the hepatocyte growth factor (HGF)/MET signaling cascade, have garnered significant attention. Notably, various studies, including our own, have highlighted the enhanced recovery of locomotor functions achieved in SCI animal models by combining HGF pretreatment and human induced stem cell-derived neural stem/progenitor cell (hiPSC-NS/PC) transplantation. However, these studies implicitly hypothesized that the functionality of HGF in SCI would be time consistent and did not elucidate its dynamics. In the present article, we investigated the time-course of the effect of HGF on SCI, aiming to uncover a more precise mechanism for HGF administration, which is indispensable for developing crystallizing protocols for combination therapy. To this end, we performed a detailed investigation of the temporal variation of HGF using the RNA-seq data we obtained in our most recent study. Leveraging the time-series design of the data, which we did not fully exploit previously, we identified three components in the effects of HGF that operate at different times: early effects, continuous effects, and delayed effects. Our findings suggested a concept where the three components together contribute to the acceleration of neurogenesis and immunomodulation, which reinforce the legitimacy of empirically fine-tuned protocols for HGF administration and advocate the novel possibility that the time-inconsistent effects of HGF progressively augment the efficacy of combined therapy.

**Supplementary Information:**

The online version contains supplementary material available at 10.1186/s41232-024-00322-9.

## Background

Human induced stem cell (hiPSC)-derived neural stem/progenitor cells (hiPSC-NS/PCs) have served as invaluable tools for investigating the intricate molecular dynamics of developing human brains *in vitro* [1, 2]. In addition to their role in models, these cells also have potential as powerful materials in regenerative therapy for the central nervous system (CNS), which is considered uncurable once it is damaged. For the clinical application of hiPSC-NS/PCs in regenerative medicine in the CNS, it is crucial to elucidate the mechanism of neuronal development and engineer an effective approach for inducing differentiation [2, 3]. In our prior studies, we identified γ-secretase inhibitors (GSIs) as key compounds that trigger the neuronal differentiation of neural stem cells (NSCs) and elucidated the underlying mechanism [4]. In addition to in vitro experiments, we have focused on developing therapies for spinal cord injury (SCI) via hiPSC-NS/PC transplantation, and GSI treatment has been adopted in the protocols of preclinical studies [5, 6], which led to our latest advancement—the first human clinical trial of hiPSC-NS/PC transplantation in subacute SCI patients [3]. However, several studies have suggested that there is a negative correlation between the prognosis of NS/PC transplantation and the severity of the injury [7]. Moreover, cytotoxic environments, notably inflammatory responses, threaten to block neural regeneration by compromising the survival of transplanted cells [8, 9].

Using various animal models, previous studies have identified hepatocyte growth factor (HGF) as a desirable substance that induces the recovery of motor function after SCI by mediating neurogenesis, neuroprotection, angiogenesis and inflammatory responses [10–12]. In our latest report, we showed that the combination of HGF pretreatment and hiPSC-NS/PC transplantation enhanced locomotor functional recovery in a rat model of SCI [13], indicating the potential of HGF in SCI therapy. HGF is a growth factor that promotes tissue regeneration via the MET receptor [13–16]. HGF/MET signaling stimulates various signal transduction pathways, such as the SRC/focal adhesion kinase (FAK) pathway, the p120/signal transducer and activator of transcription (STAT) 3 pathway, the phosphoinositide-3 kinase (PI3K)/Akt pathway, and the Ras/MEK pathway [17]. These pathways contribute to the reported effects of HGF on immunomodulation, cell proliferation, and neuronal differentiation [13, 16, 18–21]. In our previous report, we analyzed RNA-seq data from HGF-treated rat SCI models and controls. These models were sacrificed at two distinct time points to demonstrate the favorable effects of HGF on neuronal differentiation. However, we did not perform timewise comparisons in that study, as investigating the temporal variation in the effect of HGF was not our focus at that time. A longitudinal assessment of these data is now considered crucial, as it would not only reveal whether the effects of HGF are consistent over time but also provide deeper insights into the mechanism of HGF. These findings are particularly essential for developing combination therapy comprising HGF pretreatment and hiPSC-NS/PC transplantation since expanding our knowledge will refine and optimize HGF pretreatment protocols.

In the present study, we utilized the RNA-seq data from our previous study to determine whether the effects of HGF change over time. However, the changes in HGF-related effects over time and in the injured spinal cord are closely related. HGF/MET signaling serves as a hub for downstream cascades, and HGF evokes diverse functional changes in cells depending on the surrounding context. Where samples exhibit temporal changes, observers may struggle to discern whether alterations are due to the passage of time or changes in the effect of HGF. When comparing samples with or without HGF at several time points, the interdependence of sample changes and the effects of HGF complicate differentiation. To address this, we initiated our investigation by verifying the influence of HGF on the progression of injury to the spinal cord.

## Results

### The two axes: the timewise trajectory of SCI and the function of HGF

**Fig. 1:**
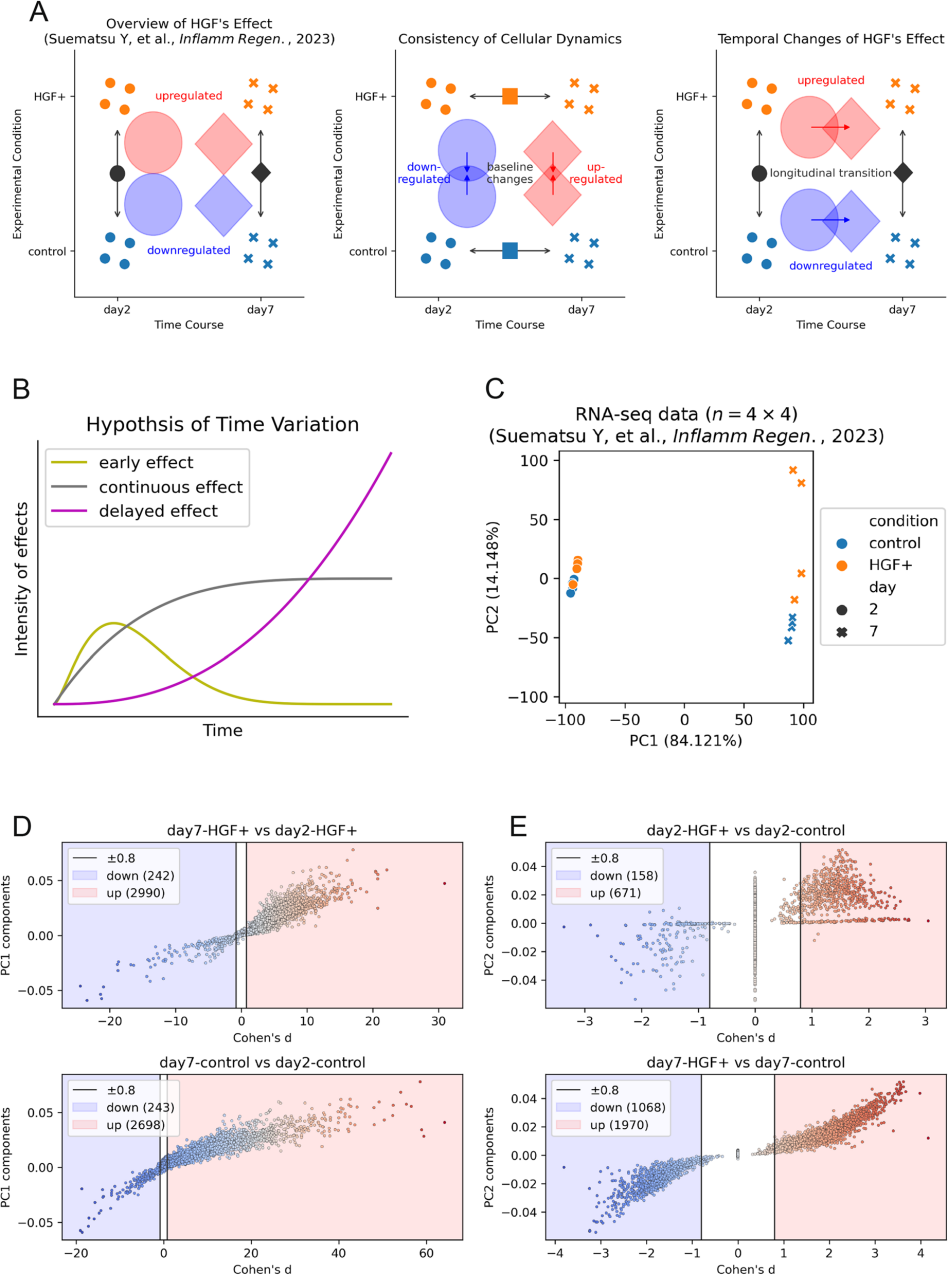
The two pivotal foci of this study: the time axis and the effect of HGF. **A** Schematics illustrating the scopes of the comparative analyses. Suematsu et al. provided an overview of the effect of HGF across a time series (left). To explore its dynamics along the time axis, we conducted “multidimensional” comparisons. First, we identified upregulated (or downregulated) genes in terms of the comparative factor (X) and quantified the overlaps of those genes with the other factor (Y) using Venn diagrams. This procedure enabled us to visualize the degree of similarity (or difference) in terms of X between the two series that are different in Y. We measured the overlap of timewise upregulation/downregulation between HGF + and control samples to juxtapose their timewise trajectories (center) and the overlap of gene regulation driven by HGF on Days 2 and 7 to visualize the temporal shifts in the HGF effect (right). **B** Conceptual schematic for the temporal variation in the effect of HGF. In addition to some time-consistent effects (denoted as continuous effects), effects that appear temporarily in the early stages (denoted as early effects) and effects that surge in the later stages (denoted as delayed effects) can be considered. **C** Overview of the dataset: The marker colors reflect the status of HGF administration, while the marker shapes indicate the time points. Each axis is a PC with a labeled contribution rate. **D** Scatter plots of genes (horizontal axis: Cohen’s d values; vertical axis: PC1 components) for HGF + (top) and control (bottom) rats. We defined the upregulated genes as those with a Cohen’s d > 0.8 and the downregulated genes as those with a value < 0.8. Note that the upregulated genes were enriched in Day 7 samples, and the vertical axis was not related to gene selection (for visualization purposes only). **E** Scatter plots of genes (horizontal axis: Cohen’s d values; vertical axis: PC2 components) for Day 2 (top) and Day 7 (bottom). The same definitions of the upregulated/downregulated genes as in (D) were applied. Note that the upregulated genes were enriched in HGF + samples, and the vertical axis was for visualization purpose only

Before enumerating the results, we will clarify what was observed in the RNA-seq data analysis conducted in our previous work [13] and reiterate our specific aims for this study. In our past study, we generated severe contusion SCI models immediately followed by continuous intrathecal administration of recombinant human HGF (or PBS for control) for either two days or seven days (Fig. S1A), and RNA-seq data from those samples demonstrated that HGF enhanced neurogenesis-related Gene Ontology (GO) terms and suppressed inflammation-related GO terms at both Day 2 and Day 7 [13]. Although we observed slight differences in the detailed composition of inflammatory GO terms at Days 2 and 7, semantically similar terms were identified that promote neurogenesis and suppress the inflammatory response, fibrosis, and gliosis [13]. Since the RNA-seq data analysis was only a part of our previous study (Suematsu et al. 2023), which aimed to elucidate the efficacy of the combined therapy of hiPSC-NS/PC transplantation and prior HGF administration, with the goal of providing an overview of the effects of HGF (Fig. 1A), the possibility of temporal variation in the effect of HGF was not considered. Even modeling the state transition of spinal cords as discrete for simplicity, HGF’s effect can be considered as a function that maps one state to another, hence, samples’ reactions to HGF (i.e., the pair of the original state and the mapped state) can differ depending on the current states of the samples (Fig. S1B). Therefore, HGF can have different effects on SCI samples depending on time points (e.g., early effects, continuous effects, and delayed effects in Fig. 1B) unless the effect of HGF is singular and steady (as implicitly hypothesized in previous studies). To provide a time-resolved description of the mechanism of HGF, we first assessed whether the SCI samples underwent similar transcriptomic transitions independent of HGF (otherwise, the time course and the effect of HGF would not be separable in this dataset); then, we examined whether the effect varied over time (Fig. 1A). Using an analogy of vectors that deviates somewhat from mathematical rigor for the sake of intuitive explanation, we considered RNA-seq data as a vector space with genes as the basis vectors, where orientations correspond to biological semantics. This interpretation is inspired by GO terms, which convey abstract directions of biological functions with collective genes. Given this analogy, our goals can be reformulated as follows: 1) to test whether the effect of time on the control samples ${\varvec{t}}$ and the HGF-treated (HGF +) samples ${{\varvec{t}}}^{\boldsymbol{*}}$ shares a common directionality (i.e., ${{\varvec{t}}}^{\boldsymbol{*}}=\alpha {\varvec{t}}$ where $\alpha$ is a scalar, referring to ${\varvec{t}}\propto {{\varvec{t}}}^{\boldsymbol{*}}$); and 2) if the effect of HGF on Day 2 and Day 7 (${\varvec{h}}\left(2\right)$ and ${\varvec{h}}(7)$, respectively) shows collinearity (i.e., ${\varvec{h}}(7)=\beta {\varvec{h}}(2)$ where $\beta$ is a scalar, referring to ${\varvec{h}}(2)\propto {\varvec{h}}(7)$). Representing the Day 2 control samples as ${\varvec{C}}$, the Day 7 HGF + samples can be denoted as ${\varvec{C}}+{\varvec{h}}\left(2\right)+{{\varvec{t}}}^{\boldsymbol{*}}$ and ${\varvec{C}}+{\varvec{t}}+{\varvec{h}}(7)$ (Fig. S1C). Assuming that ${\varvec{t}}\propto {{\varvec{t}}}^{\boldsymbol{*}}$ provides insight into the temporal variation in the effect of HGF, ${\varvec{h}}\left(7\right)-{\varvec{h}}\left(2\right)=\left(\alpha -1\right){\varvec{t}}\propto {\varvec{t}}\propto {{\varvec{t}}}^{\boldsymbol{*}}$ will be derived from ${\varvec{C}}+{\varvec{h}}\left(2\right)+{{\varvec{t}}}^{\boldsymbol{*}}={\varvec{C}}+{\varvec{t}}+{\varvec{h}}(7)$ (without this assumption, ${\varvec{h}}\left(7\right)-{\varvec{h}}\left(2\right)={{\varvec{t}}}^{\boldsymbol{*}}-{\varvec{t}}$ remains unsolvable). Hence, we decided to consider ${\varvec{t}}$ and ${{\varvec{t}}}^{\boldsymbol{*}}$ before ${\varvec{h}}\left(7\right)-{\varvec{h}}\left(2\right)$, our primary goal, to take advantage of the structural simplicity derived from ${\varvec{t}}\propto {{\varvec{t}}}^{\boldsymbol{*}}$. We reiterate that our conceptualization involves an analogy to vectors, where we deliberately sacrifice some mathematical rigor for the sake of providing an intuitive explanation. In the Supplemental Information, we provide detailed descriptions of these vectors and include clarifying statements for the hypotheses introduced with occasionally ill-defined notations. Regarding practical method of comparisons, we utilized Venn diagrams to visualize the overlaps in sets of upregulated/downregulated genes for different experimental conditions (Fig. 1A). Further details on this scheme will be explained later.

In the first step of the data analysis, we visualized the dataset via principal component analysis (PCA) of the logarithmic-transformed transcript per million (TPM) data (Fig. 1C), and we filtered out upregulated and downregulated genes for each case by setting a threshold of $\pm$ 0.8 for Cohen’s d values, as this parameter indicates that the effect size of the difference in means between the two given conditions is large [22]. Given that homoscedasticity among all four conditions was not assured for all genes, we adopted Cohen’s d value instead of fold change as an indicator of upregulation/downregulation because it can balance the means and the standard deviations. Cohen’s d values were displayed along with the components (i.e., eigenvectors) of the principal components (PCs) on the vertical axes for visualization purposes (Fig. 1D,E). This presentation was adopted because PC1 appeared to align with the time axis, while PC2 seemed to correspond to the effect of HGF (Fig. 1C). The top 15 and the bottom 15 genes according to the PC1 (and/or PC2) component values are shown in Fig. S1D for reference. Longitudinal comparisons were made between Day 2 and Day 7 to identify upregulated and downregulated genes, respectively, in both the HGF + and control groups (Fig. 1D). Cross-sectional comparisons between HGF + and control cells were also conducted at the indicated time points (Fig. 1E). The first set of comparisons aimed to capture temporal changes in the HGF + /control groups, which was essential for assessing their similarity. In contrast, the second set included snapshots of the effect of HGF, emphasizing longitudinal variations.

### Similarities in the timewise trajectory indicated that HGF enhances neuronal development without drastically altering cell fate

Next, we evaluated whether the SCI samples exhibited similar transcriptomic trajectories irrespective of HGF administration. This was an inevitable step for evaluating the temporal variation in the effect of HGF because the assessment would be simpler if the baseline transcriptomic changes over time were the same in HGF + and control samples than if they were inconsistent.

**Fig. 2:**
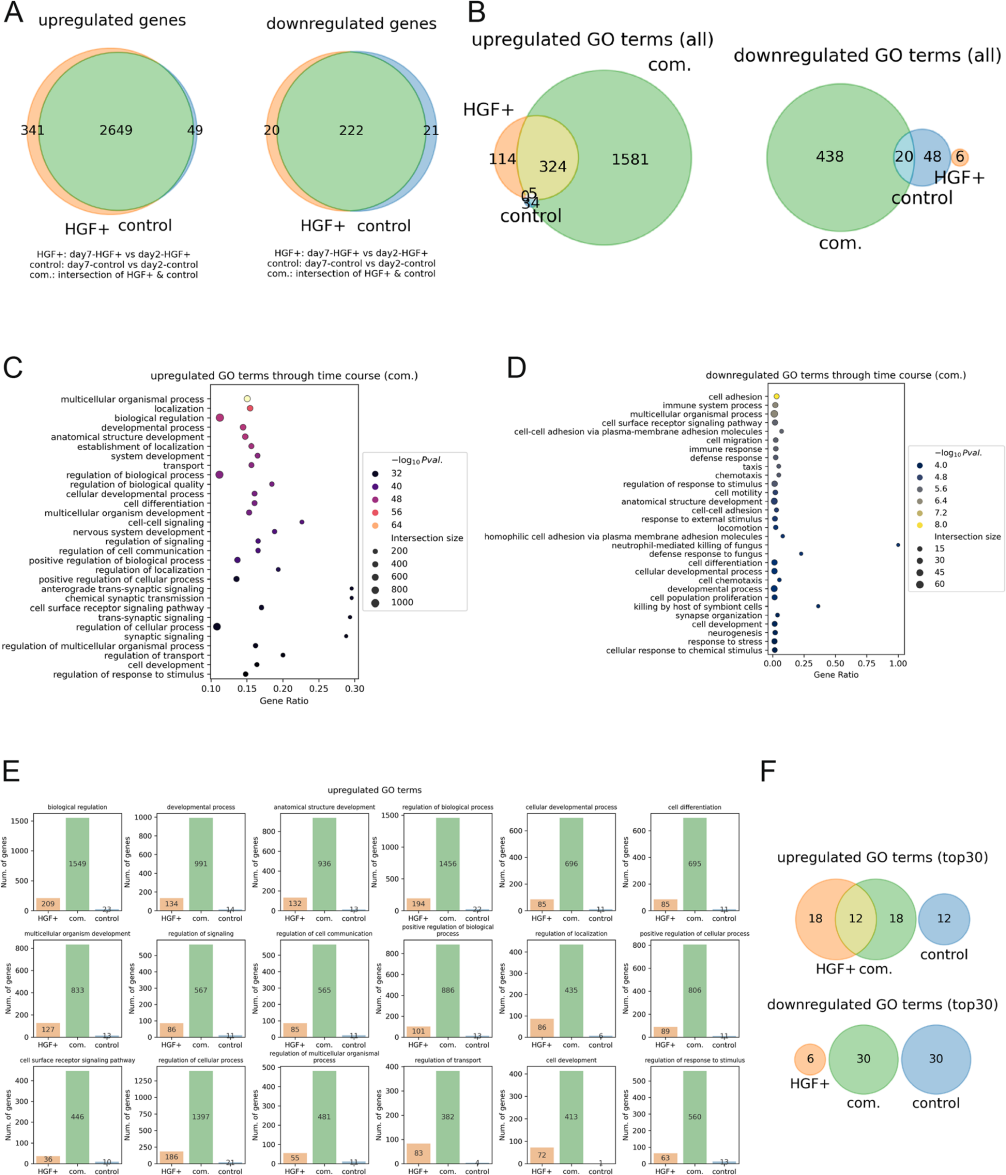
SCI samples exhibited similar trajectories regardless of HGF status. **A** Venn diagrams of upregulated genes (left) and downregulated genes (right). **B** Venn diagrams of upregulated (left) and downregulated (right) GO terms. **C**, **D** The 30 most significant GO terms for the (**C**) upregulated genes and (**D**) downregulated genes. **E** Numbers of GOTRGs in each gene subset. The top 18 significantly upregulated GO terms unique to the common gene subset (denoted as com.) were selected. **F** Venn diagrams of the top 30 upregulated GO terms (top) and downregulated GO terms (bottom)

First, we quantified the number of overlapping upregulated/downregulated genes (selected at Fig. 1D) between the HGF + group and the control group, and the intersection of the Venn diagrams contained the most genes in both categories (Fig. 2A), indicating that the majority of the transcriptional changes observed over time were shared. The results of enrichment analyses conducted on the gene subsets (Fig. 2A: HGF + , orange; common (com.), green; and control, blue) also showed that the common subset had the largest number of GO terms (Fig. 2B), consistent with the results in Fig. 2A.

The 30 most significant GO terms (Fig. 2C–D) suggested that a variety of functions, including nervous system development, were promoted and that a wide range of processes, such as the immune response, were suppressed in the baseline dynamics of the SCI models. The 30 most significant GO terms for other subsets of upregulated/downregulated genes were also provided for reference (Fig. S2A–D). These results indicated the presence of assistive GO terms (e.g., “synaptic signaling” in the upregulated GO terms) in the HGF + gene subset, which could be considered to work in harmony with genes in the common subset. In contrast, the existence of adversarial GO terms (e.g., “immune response” in the upregulated GO terms and “trans-synaptic signaling” in the downregulated GO terms) was evident in the control gene subset, suggesting a counteraction toward the GO terms of the common subset.

Given that there are aids and interventions for baseline temporal reactions, a better understanding of these reactions helps us directly visualize the magnitude of those tendencies described in GO terms. To this end, we mapped the GO terms to the gene symbols so that we could count the numbers of GO term-related genes (GOTRGs) in the three subsets of genes, HGF + , com., and control. We identified the 18 GO terms that are unique to the com. subset and the number of corresponding GOTRGs (Fig. 2E); these 18 GO terms corresponds to the green region of the upper Venn diagram of Fig. 2F and are components of the top 30 significantly upregulated GO terms (Fig. 2C). As shown in Fig. 2E, the com. subset had the largest number of GOTRGs for all 18 GO terms compared to the other subsets, which suggested that the contribution of HGF + administration to the common GO terms was supplemental and that the GOTRGs responsible for the baseline shifts were inclusively observed in both the HGF + and control samples. Here we emphasize that the numbers of GOTRGs were by far the greatest in com. for all 18 GO terms, which results are not self-evident even if the GO terms were selected exclusively from the set of upregulated GO terms for com. when we take the structural complexity of the relationships of gene symbols and GO terms accounted. Gene symbols and GO terms are completely different domains and their elements are intertwined in many-to-many relations, and enrichment analysis scoops GO terms that seem statistically likely for the given set of the gene symbols (i.e., DEGs) while other GO terms tagged with those gene symbols can be ignored. Therefore, there are two possibilities that can be unintuitive: 1) some DEGs that play various roles in multiple contexts can be tagged with GO terms that seems intuitively irresponsible; and 2) some GO terms can be identified for different DEG groups. Given those pitfalls, we believe it critical to double-check the GO terms with the number of GO terms and the number of GOTRGs for summarizing biological phenomena behind the samples. This time, it is natural that com. has the largest number of GOTRGs for GO terms suggested exclusively in the genes in com., yet the existence of drastic gaps in all 18 GO terms is still noteworthy. As the interpretations from Fig. 2A, B, E do not contradict to each other, we could conclude that the dominant biological reactions taken place along with the time-course were quite common regardless of HGF. For additional information, the top 30 significantly downregulated GO terms were also associated with the downregulated genes in the com. subset (Fig. S2E), which indicated that the baseline suppression was almost consistent regardless of HGF administration.

In summary, although HGF partially assisted in baseline temporal reactions, these changes were almost independent and commonly observed in HGF + and control samples. In other words, we could assume that almost the same baseline timewise transcriptomic variations were observed in both the HGF + and control groups. Given these results, simple comparisons of the effect of HGF at different time points were conducted to reveal the temporal variation.

### Longitudinal observation of gene expression suggested that HGF can trigger, maintain, and reinforce neuronal differentiation over a specific time course

Next, we elucidated the temporal variation in the effect of HGF, which was overlooked in the report by Suematsu et al., by longitudinal comparisons of cross-sectional transcriptomic variations caused by HGF to highlight the difference in the effects of HGF between these two time points.

**Fig. 3:**
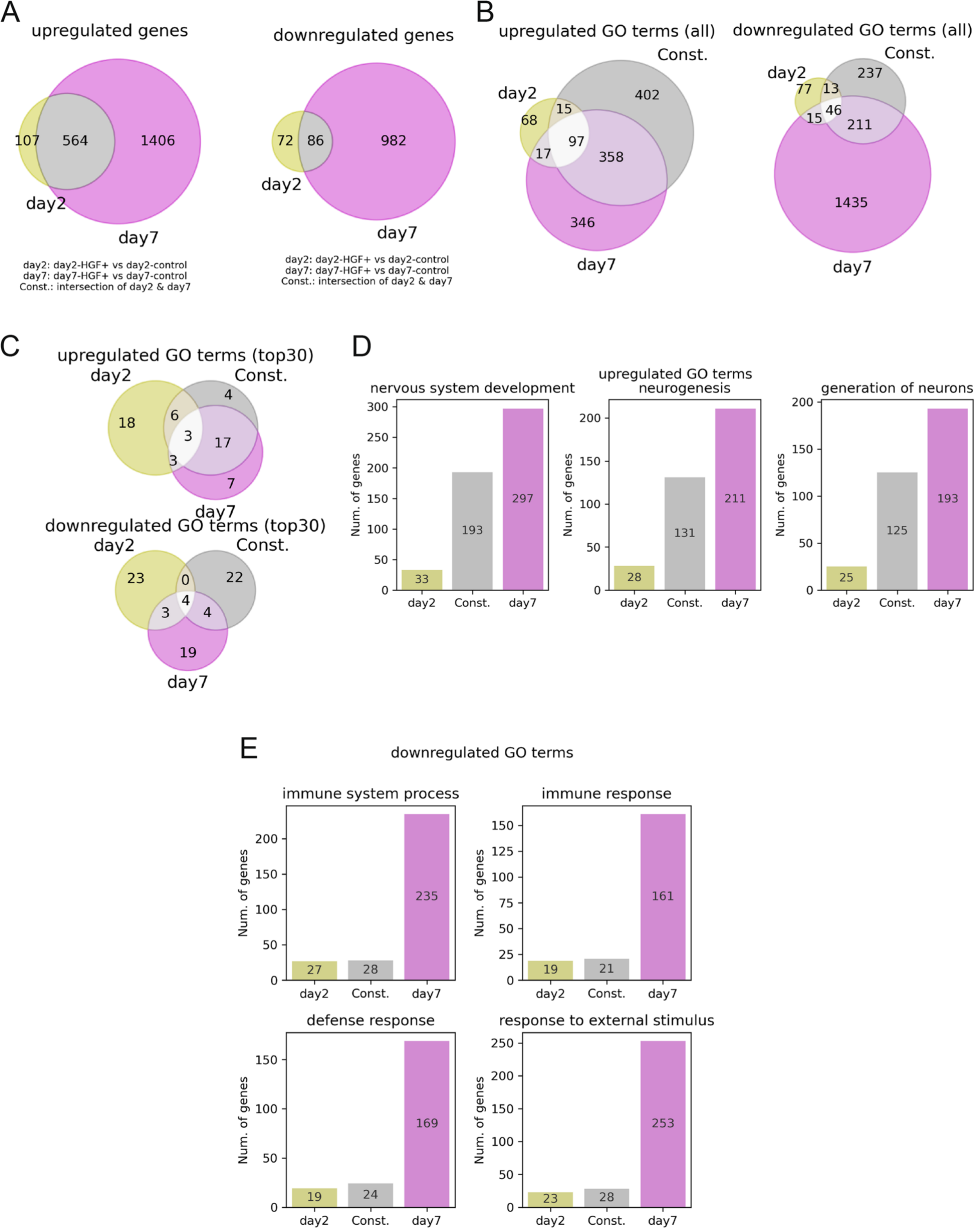
Time-resolved stratification revealed timewise shifts in the HGF effect. **A** Venn diagrams of upregulated genes (left) and downregulated genes (right). **B** Venn diagrams of upregulated GO terms (left) and downregulated GO terms (right). **C** Venn diagrams of the top 30 upregulated GO terms (top) and downregulated GO terms (bottom). **D–E** Numbers of GOTRGs in each gene subset. The three GO terms at the intersection of Day 2, constant (denoted as Const.), and Day 7 in the Venn diagram of upregulated GO terms were selected for (**D**); likewise, the four GO terms from the Venn diagram for downregulated GO terms were selected for (**E**)

Like in the previous section, we quantified the number of overlapping upregulated/downregulated genes (selected in Fig. 1E) between Day 2 and Day 7. While the com. subset had the most upregulated and downregulated genes, as shown in Fig. 2A, the proportions of elements were relatively even for each subset (Day 2, yellow; Const., gray; and Day 7, magenta in Fig. 3A). This indicated the existence of three major effects of HGF: early effects were exclusively observed on Day 2, continuous effects occurred constantly from Day 2 to Day 7, and delayed effects ultimately appeared on Day 7. The Venn diagrams of the GO terms supported this concept, as they had nonnegligible numbers of elements in each section (Fig. 3B–C). Although one might question the subtleness of the boundary between the state where a single subset is dominant and the state where multiple subsets have sufficient elements, it is crucial to emphasize that the Const. subset was not very dominant, which indicated that the effect of HGF was not consistent across time. Hence, these results affirmed the time-varying aspects of the effect of HGF.

Next, we identified the top 30 significant GO terms for Day 2, Const., and Day 7 (Fig. S3A–F). These results did not contradict those of Suematsu et al.; neurogenesis-related GO terms were upregulated, and inflammation-related GO terms were downregulated. To elucidate the mechanism of HGF through a time-resolving analysis and establish a logical connection between our concepts of time variation and Suematsu et al.’s in vivo experiments, we investigated the most prominent effects of HGF on SCI samples. Therefore, we considered the GO terms at the innermost intersection of the Venn diagram depicting the top 30 significant GO terms (Fig. 3C–E). The numbers of GOTRGs in the gene subsets showed consistent trends, with the lowest numbers occurring on Day 2 and increasing incrementally until Day 7 (Fig. 3D–E). These findings suggested that a limited number of genes triggered the reaction of HGF as an early effect, an intermediate number of genes contributed to maintaining a continuous effect, and a population of genes reinforced them as a delayed effect.

## Discussion

In this study, we conducted multidirectional comparative analyses on two occasions, revealing two cardinal findings. First, we identified a consistent pattern of basal temporal variations in SCI samples, irrespective of HGF treatment status. Second, we identified the commonality of basal temporal variations in SCI samples across different HGF administration statuses and the time-varying aspects of the effect of HGF based on the concept that different genes exhibit activity at different times. Our study revealed that the impact of HGF, which was also observed by Suematsu et al. [13], is significantly enhanced by its delayed effects. Consequently, administering HGF for a period of 7 days may be more effective than a 2-day regimen. We found that some genes were consistently upregulated on both Day 2 and Day 7, which was consistent with the findings of our prior study. In contrast, we identified a smaller subset that was initially upregulated on the second day but did not maintain elevated expression levels through the seventh day. Furthermore, we observed a larger set of genes whose expression was upregulated specifically on Day 7, suggesting a delayed response. These findings might indicate that the initial upregulation of the smaller subset triggers subsequent upregulation in the larger set, resulting in a delayed positive effect. This discovery, previously unreported, highlights the intricate dynamics uncovered in our research.

Our findings may provide further insight not only into SCI but also into several other neurological disorders regulated by MET and its downstream signaling cascades. For example, while the association between MET and schizophrenia was reported in a genome-wide association study (GWAS) [23] in 2010, in the same year, Cannon criticized the results for their inconsistency with other GWAS reports, suggesting the potential false positivity of the correlation between MET and schizophrenia due to loss of statistical power [24]. In addition, Cannon emphasized that the insufficiency of co-occurrence between MET and lower intellectual functioning supports its unrelatedness to the etiopathophysiology of schizophrenia. Although our findings would not directly address these respects, the dynamic changes in the effect of HGF are noteworthy, especially if the fluctuations in its effects might regulate pivotal genes involved in pathogenesis. By aligning the incremental range of the downstream gene regulation of HGF with elapsed time, longitudinal stratification of certain pathogenic events (e.g., intracranial inflammation) might help identify the subtle causality of schizophrenia-related gene regulation due to the absence of time-varying effects of HGF/MET signaling. Given that neuroinflammation and immune dysregulation and their link to schizophrenia have long been discussed [25] and that there is some evidence that indicates that postnatal/childhood infections induce schizophrenia by interfering with CNS development [26–28], there may be an overlap between temporal shifts in HGF-MET signaling effects on neurodevelopment and schizophrenia.

Furthermore, our findings would provide another hint for studies trying to reveal the roles of inflammatory cytokines in neurogenesis. The involvement of inflammation to neurogenesis, especially adult neurogenesis, is widely discussed across different diseases [29] such as Alzheimer’s disease [30, 31], depression [32], epilepsy [33], subarachnoid hemorrhage [34], and also SCI [7–9, 13], where neuroinflammation intervenes neurogenesis mediated by glial- or microglial-activities and those functions are closely related to either pathological mechanisms or therapeutical bottlenecks [29, 35]. There are various inflammatory cytokines reported to assist or antagonize neurogenesis [29], hence, roles of inflammatory response in neurogenesis might not be a black-and-white situation. As our concept to introduce the time-series transition to cellular states and functionality of molecules are built with results from multi-dimensional comparisons to decompose the effect of mediator molecules and time-series, it might provide more complicated perspectives of the immune systems and neurogenesis when our scheme is adopted to studies of single-cell-level resolution.

Finally, our methodology of longitudinal analyses of cross-sectional transcriptomic data can be applied to any research investigating the temporal effects of specific treatments. The duration of drug administration for SCI is usually determined by the assessment on phenotypes, although other factors, such as dose or route, might affect the outcome. Although we have conducted several studies applying HGF in SCI therapy, there is no evidence for the optimal duration of HGF administration, and the protocols have been empirically fine-tuned. In our latest study in which we demonstrated the efficacy of HGF pretreatment prior to hiPSC-NS/PC transplantation, we determined the time points at which to sacrifice the rats according to previous reports. In the present study, we introduced an innovative approach comprising multidirectional comparisons of gene regulation patterns and evaluation of their similarity through Venn diagrams. This methodology allowed us to present a brief proof of concept for secondary effects activated subsequent to the primary effect. Our findings enhance the credibility of the empirical HGF administration protocol by providing evidence that this delay contributes positively to therapeutic progress and suggesting that a seven-day administration period is more beneficial than a two-day period. It is worth mentioning that our study does not aim deterministic optimization of the protocol, therefore, the appropriate parameter search space is vaster than what we observed. While we used RNA-seq data from our previous study in which rats were sacrificed at two time points after a state-of-the-art HGF administration protocol, our investigation could not extend beyond Day 7, when potential positive or negative nth-order effects on SCI might occur. Nevertheless, our study provides valuable contributions by proposing a solution for the complete black-box process of optimizing the duration of HGF administration.

## Methods

### Data preprocessing

We concatenated two separate raw data Excel files shared by Suematsu et al. into a single file, replaced the not-a-number (NaN) values with zeros, and performed a logarithmic transformation on the TPM values so that the output matrix consisted of only the log_2_(TPM + 1) values. We also generated a metadata table from the Excel sheets. As we intended to avoid managing the data files on GitHub, we also implemented a function to load the formatted data/metadata files. Hence, readers interested in reproducing our analyses are encouraged to refer to our repository.

### PCA

PCA was conducted using scikit-learn [36]. The PCA components (i.e., the eigenvectors of each PC) were also calculated with the Scikit-learn implementation.

### Selection of upregulated/downregulated genes

Cohen’s d value between sample populations $X$ and $Y$ for a statistical variable can be defined as the equation below [22], in which the mean values for the populations are denoted as ${M}_{X}$ or ${M}_{Y}$, the sample standard deviations are ${s}_{X}$ or ${s}_{Y}$, and the sample sizes are ${n}_{X}$ or ${n}_{Y}$:

$$d:=\frac{{M}_{X}-{M}_{Y}}{\sqrt{\frac{{n}_{X}{s}_{X}^{2}+{n}_{Y}{s}_{Y}^{2}}{{n}_{X}+{n}_{Y}}}}$$

Setting the threshold to $\pm$ 0.8, upregulated genes and downregulated genes were identified. In detail, Day 7 was treated as population $X$ and Day 2 as $Y$ in Fig. 1D, and HGF + was treated as $X$ and the control as $Y$ in Fig. 1E. Therefore, genes that were more highly expressed in population $X$ would have positive d values, while other genes that were expressed in population $Y$ would have negative d values. Genes with d values > 0.8 were classified as upregulated genes, and those with d values < 0.8 were classified as downregulated genes.

### Enrichment analysis and identification of GOTRGs

Enrichment analysis was conducted with gprofiler2 [37], and the results were visualized using Matplotlib [38] and Seaborn [39]. We used org.Rn.eg.db [40] to count genes that were tagged with specific GO terms and visualized the results with Matplotlib and Seaborn.

### Supplementary Information

**Supplementary Material 1.**

## Data Availability

RNA-seq data are publicly available in the Gene Expression Omnibus (GEO), and no primary data were obtained in this research. For further details on the RNA-seq data, please refer to the original article. All codes used in the data analysis have been deposited in GitHub (https://github.com/yo-aka-gene/takemura_hgf).

## Abbreviations

CNS: Central nervous system
FAK: Focal adhesion kinase
GO: Gene Ontology
GOTRG: GO term-related gene
GSI: γ-Secretase inhibitor
GWAS: Genome-wide association study
hiPSC: Human induced pluripotent cell
hiPSC-NS/PC: HiPSC-derived neural stem/progenitor cell
NaN: Not a number
NSC: Neural stem cell
PC: Principal component
PCA: Principal component analysis
PI3K: Phosphoinositide-3 kinase
SCI: Spinal cord injury
STAT3: Signal transducer and activator of transcription 3
TPM: Transcripts per million
