# Supplementary material for: Chronological transitions of hepatocyte growth factor treatment effects in spinal cord injury tissue
Source: Inflamm Regen. 2024 Mar 13;44:10. doi: 10.1186/s41232-024-00322-9 (PMC10935783; doi:10.1186/s41232-024-00322-9)
Supplement: Supplementary file 1 — Supplementary Material 1. [file 41232_2024_322_MOESM1_ESM.pdf]

Supplemental Information

Analogical explanation of our analysis design using vectors

To provide intuitive insights into our analysis design and to demonstrate why we conducted the comparison between the timewise trajectories of control and HGF+ samples (despite our primary focus on the temporal variation of the effect of HGF), we formulated the following hypotheses:

- RNA-seq data are a matrix composed of vectors from vector space $V \coloneqq \mathbb{R}^d$, where the d genes form the basis vectors ($d \in \mathbb{N}$, $\mathbb{R}$ is the set of real numbers, and $\mathbb{N}$ is the set of natural numbers). We applied this statement because it has been widely used in recent biological studies. PCA is a prominent example of an operation that requires data to constitute a vector space; it performs eigenvalue decomposition of the covariance matrix of the data according to the algebraic structures of Euclidean space (a real finite-dimensional vector space with the Euclidean norm)[1,2].
- The experimental factors can be denoted as vectors in V, allowing for linear additivity. Design of experiments is a statistical methodology that involves analyzing multiple factors, and various experimental designs, such as factorial designs and orthogonal arrays (Taguchi designs), have been developed to ensure the orthogonality of experimental factors—meaning that they exhibit no correlations and are linearly

additive[3,4]. Suematsu et al. employed a 2x2 factorial design (also known as the L4 orthogonal array) for the RNA-seq data. Therefore, we can assume that the main effects of the factors (the time course and HGF administration) are orthogonal.

- Vector orientations and magnitudes intuitively correspond to either qualitative or quantitative aspects of RNA-seq data. This interpretation is inspired by GO terms, which convey abstract directions of biological functions with qualitative information (gene symbols) on collective genes. Qualitative information is a regular format for RNA-seq data analysis outputs (e.g., differentially expressed genes). Hence, this hypothesis allows us to analogize qualitative information with orientation in the realm of vector space V.
- The state where two samples are biologically similar is equivalent to the state where their corresponding vectors are collinear (i.e., a scalar $k \in \mathbb{R}$ such that $\boldsymbol{x} = k\boldsymbol{y}$ exists for vectors $\boldsymbol{x}$ and $\boldsymbol{y}$). This assumption allows us to conceptualize the analysis for validating biological similarities using the analogy of vectors. As we hypothesized that the qualitative states of RNA-seq samples can be represented as orientations of vectors, we prefer to define semantical similarities as the parallelism of vectors (more precisely, $\boldsymbol{x} = k\boldsymbol{y}$ with a positive scalar k for corresponding vectors $\boldsymbol{x}$ and $\boldsymbol{y}$ rather than $\boldsymbol{x} = \boldsymbol{y}$) because the numerical aspects (magnitudes of the vectors)

vanish during the qualitative assessment of RNA-seq data. Again, we emphasize that the aim of these assumptions is to provide clear-cut explanations for our analysis goals rather than to introduce mathematical notation itself.

Under the assumption of those properties, we denote the vectors in V corresponding to the respective experimental conditions (while excluding stochastic errors for simplicity). Denoting the Day 2-control samples as $\boldsymbol{C} \in V$ and the effect of the time course on the control samples as $\boldsymbol{t} \in V$, the Day 7-control samples can be denoted as $\boldsymbol{C} + \boldsymbol{t}$. Moreover, defining the vector function $\boldsymbol{h}: \mathbb{N} \to V$ to denote the effect of HGF on an arbitrary day $n \in \mathbb{N}$ as $\boldsymbol{h}(n)$, the Day 2-HGF+ samples was denoted as $\boldsymbol{C} + \boldsymbol{h}(2)$. Considering the effect of the time course on the HGF+ samples as $\boldsymbol{t}^* \in V$, the Day 7-HGF+ samples can be denoted as $\boldsymbol{C} + \boldsymbol{h}(2) + \boldsymbol{t}^*$ and $\boldsymbol{C} + \boldsymbol{t} + \boldsymbol{h}(7)$. Given that, the relationship between $\boldsymbol{h}(2)$ and $\boldsymbol{h}(7)$ can be denoted as follows:

$$\boldsymbol{h}(7) = \boldsymbol{h}(2) + \boldsymbol{t}^* - \boldsymbol{t} \Leftrightarrow \boldsymbol{h}(7) - \boldsymbol{h}(2) = \boldsymbol{t}^* - \boldsymbol{t}.$$

(Eq. 1)

In this study, our objectives were to validate 1) whether the control/HGF+ samples underwent similar transcriptomic transitions regardless of HGF administration and 2) whether the effect of HGF changed over time. Hence, we decided to quantify the intersections of upregulated (or downregulated) genes to validate the similarities between 1) the longitudinal comparisons (“Day 2-control vs. Day 7-control” and “Day 2-

HGF+ vs. Day 7-HGF+") and 2) the cross-sectional comparisons ("Day 2-control vs. Day 2-HGF+" and "Day 7-control vs. Day 7-HGF+"). Given all the initial assumptions, we can analogize the two objectives as the collinearity of $\boldsymbol{t}$ to $\boldsymbol{t}^*$ and that of $\boldsymbol{h}(2)$ to $\boldsymbol{h}(7)$. With positive scalars $\exists\alpha, \beta \in \mathbb{R}_{\geq 0}$, the following equations hold:

$$\boldsymbol{t}^* = \alpha\boldsymbol{t}$$

(Eq. 2)

$$\boldsymbol{h}(\mathbf{7}) = \boldsymbol{\beta}\boldsymbol{h}(\mathbf{2}),$$

(Eq. 3)

Note that both Eq. 2 and 3 refer to $\boldsymbol{t} \propto \boldsymbol{t}^*$and $\boldsymbol{h}(2) \propto \boldsymbol{h}(7)$), respectively.

To illustrate the advantage of incorporating Eq. 2 into Eq. 1, let us consider a scenario where $\boldsymbol{t}$ and $\boldsymbol{t}^*$ are entirely different vectors, rendering Eq. 2 inapplicable. While our primary focus is to validate the time variation in the effect of HGF ($\boldsymbol{h}(7) - \boldsymbol{h}(2)$), Eq. 1 becomes unsolvable under this assumption. Consequently, $\boldsymbol{h}(7) - \boldsymbol{h}(2)$ and $\boldsymbol{t}^* - \boldsymbol{t}$ become indistinguishable. Even if Eq. 3 (which refers to the time consistency of the effect of HGF) holds, the presence of $\boldsymbol{t}^* - \boldsymbol{t}$ persists in the equation. Given that $\boldsymbol{t}^* - \boldsymbol{t}$ can be influenced by the effect of HGF from Day 2 through Day 7, the discussion on $\boldsymbol{h}(7) - \boldsymbol{h}(2)$ becomes intricate, entangled in circular arguments involving the effect of HGF and $\boldsymbol{t}^* - \boldsymbol{t}$; the discourse on $\boldsymbol{h}(7) - \boldsymbol{h}(2)$ relies on $\boldsymbol{t}^* - \boldsymbol{t}$, which, in turn, depends on the effect of HGF, creating mutual dependence.

In contrast, the following equation holds when Eq. 1 and Eq. 2 are valid:

$$\boldsymbol{h}(7) = \boldsymbol{h}(2) + (\alpha - 1)\boldsymbol{t} \ \Leftrightarrow\ \boldsymbol{h}(7) - \boldsymbol{h}(2) = (\alpha - 1)\boldsymbol{t}.$$

(Eq. 4)

Notably, Eq. 4 indicates that $\boldsymbol{h}(7) - \boldsymbol{h}(2)$ can be inferred to align with the canonical timewise transition of SCI samples ($\because \boldsymbol{h}(7) - \boldsymbol{h}(2) \propto \boldsymbol{t} \propto \boldsymbol{t}^*$), irrespective of other conditions, such as Eq. 3. Since the justification of Eq. 2 can be supported by the data interpretation, validating Eq. 2 provides structural simplicity to the arguments on the temporal variation of the effect of HGF.

In conclusion, we decided to compare the timewise trajectories of control and HGF+ samples prior to elucidating the temporal variation in the effect of HGF. Anticipating that the similarity in the temporal transitions of the two groups would be a pivotal factor influencing the conceptual complexity of this study, we provided a comprehensive explanation by drawing a parallel with vector spaces.

Supplemental figure legends

Figure S1. RNA-seq sample details and the concept of temporal variation

(A) Graphical explanations of the detailed information regarding the RNA-seq data. The deposited data contained 4 samples each for the four conditions (therefore 16 samples

in total): day2-control, day2-HGF+, day7-control, and day7-HGF+. As shown in the primary article, severe contusion SCI model rats were generated, immediately received continuous intrathecal administration of human recombinant HGF (or PBS for control) and were sacrificed on either Day 2 or Day 7, after which RNA-seq was performed on the dissected spinal cords. The reader is referred to the original article for additional details.

(B) Discrete state transition model of spinal cord samples (ovals) and the effect of HGF (defined as a mapping between samples of state k and state k+1, which is dependent on the current state of the sample).

(C) Analogical explanations of our analysis design using a vector space as a coordinate system of gene expressions. Supposing the experimental conditions (time and addition of HGF) affect separately to each other so that we can decompose their effects as vectors (such as time effects or effects of HGF), our interests in this paper can be rephrased as 1) checking if the effect of time course on control samples ($\boldsymbol{t}$) and that of HGF+ samples ($\boldsymbol{t}^{*}$) are similar and 2) evaluating if the effect of HGF on day2 ($\boldsymbol{h}(2)$) and that of day7 ($\boldsymbol{h}(7)$) are similar. As RNA-seq analyses frequently visualize biological similarity with semantical representations (e.g., gene names, or GO terms) rather than numerical data, we consider that those schemes can be represented as

validations of vectors to be parallel (e.g., $\boldsymbol{t} \propto \boldsymbol{t}^{*}$and $\boldsymbol{h}(2) \propto \boldsymbol{h}(7)$). Please refer to the "**Analogical explanation of our analysis design using vectors**" section in the Supplemental Information for detailed descriptions.

(D) The top 15 and bottom 15 genes of PC1 components (left) and PC2 components (right). The name "components" is an alias for the eigenvectors of PCs used in scikit-learn.

Figure S2. Further details on timewise baseline shifts in HGF+/control samples

(A–D) The 30 most significant GO terms for (A) upregulated genes for HGF, (B) downregulated genes for HGF, (C) upregulated genes for the control, and (D) downregulated genes for the control.

(E) Numbers of GOTRGs in each gene subset. The top 30 significantly downregulated GO terms uniquely found in the common gene subset (denoted as com.) were selected.

Figure S3. Details on the time-resolved effect of HGF.

(A–F) The 30 most significant GO terms for (A) upregulated genes on Day 2, (B) downregulated genes on Day 2, (C) upregulated genes for Const., (D) downregulated

genes for Const., (E) upregulated genes on Day 7, and (F) downregulated genes on Day 7.

Supplemental References

1. Shlens, J. A Tutorial on Principal Component Analysis. (2014) doi:https://doi.org/10.48550/arXiv.1404.1100.
2. Rao, C. R. The use and interpretation of principal component analysis in applied research. *Sankhyā Indian J. Stat. Ser. A* **26**, 320–358 (1964).
3. Rao, R. S., Kumar, C. G., Prakasham, R. S. & Hobbs, P. J. The Taguchi methodology as a statistical tool for biotechnological applications: A critical appraisal. *Biotechnol. J.* **3**, 510–523 (2008).
4. Beder, J. H. *Linear Models and Design*. *Linear Models and Design* (Springer International Publishing, 2022). doi:10.1007/978-3-031-08176-7.

Fig. S1

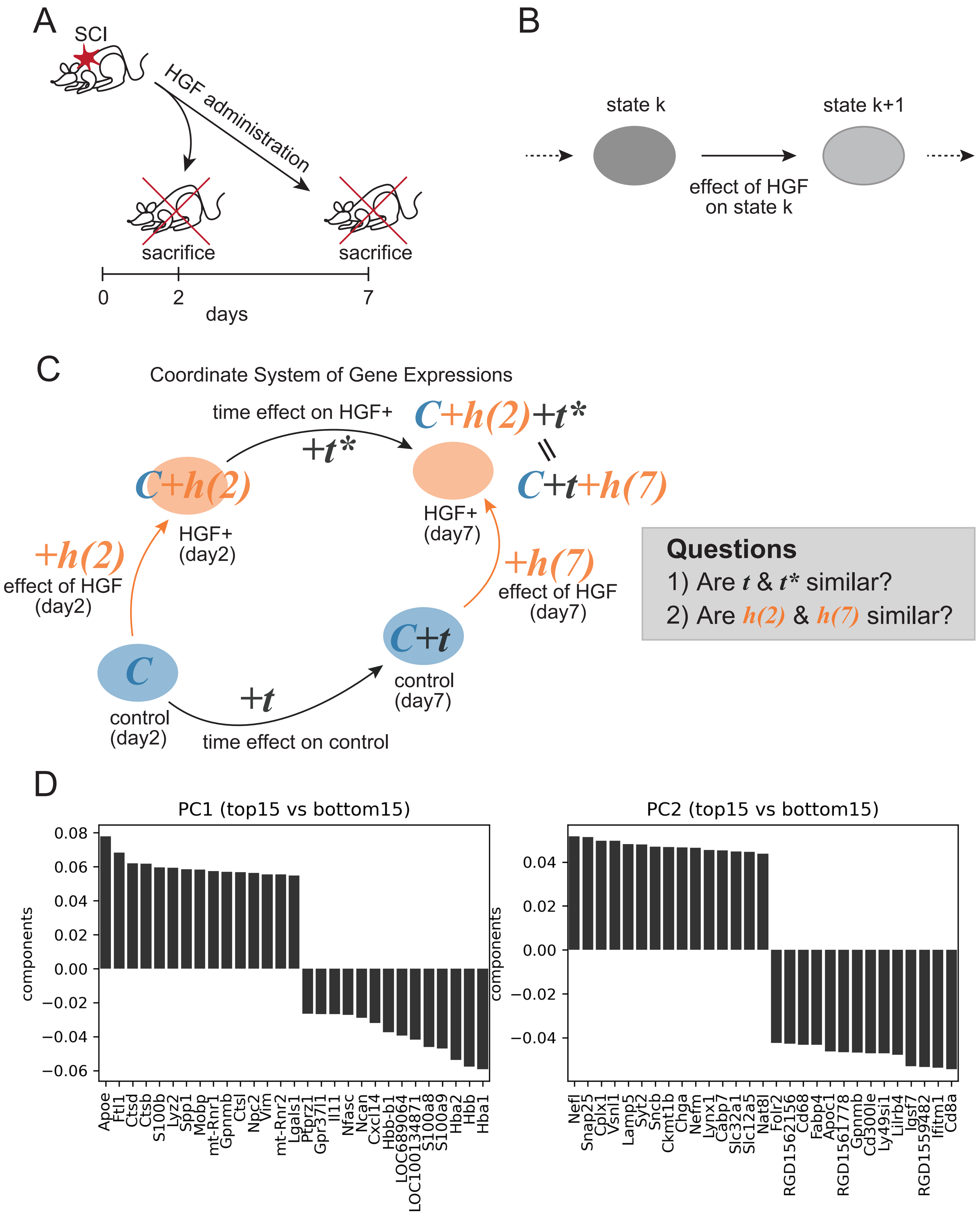

A
SCI
HGF administration
sacrifice
sacrifice
0
2
7
days
B
state k
state k+1
effect of HGF
on state k
C
Coordinate System of Gene Expressions
time effect on HGF+
+t*
C+h(2)+t*
=
C+t+h(7)
C+h(2)
HGF+
(day2)
HGF+
(day7)
+h(2)
effect of HGF
(day2)
+h(7)
effect of HGF
(day7)
C
control
(day2)
+t
time effect on control
C+t
control
(day7)
Questions
1) Are t & t* similar?
2) Are h(2) & h(7) similar?
D
PC1 (top15 vs bottom15)
PC2 (top15 vs bottom15)
components
Apoe
Ftl1
Ctsd
Ctsb
S100b
Lyz2
Spp1
Mobp
mt-Rnr1
Gpnmb
Ctsl
Npc2
Vim
mt-Rnr2
Lgals1
Ptprz1
Gpr37l1
Il11
Nfasc
Ncan
Cxcl14
Hbb-b1
LOC689064
LOC100134871
S100a8
S100a9
Hba2
Hbb
Hba1
Nefl
Snap25
Cplx1
Vsnl1
Lamp5
Syt2
Sncb
Ckmt1b
Chga
Nefm
Lynx1
Cabp7
Slc32a1
Slc12a5
Nat8l
Folr2
RGD1562156
Cd68
Fabp4
Apoc1
RGD1561778
Gpnmb
Cd300le
Ly49si1
Lilrb4
Igsf7
RGD1559482
Ifitm1
Cd8a

Fig. S2

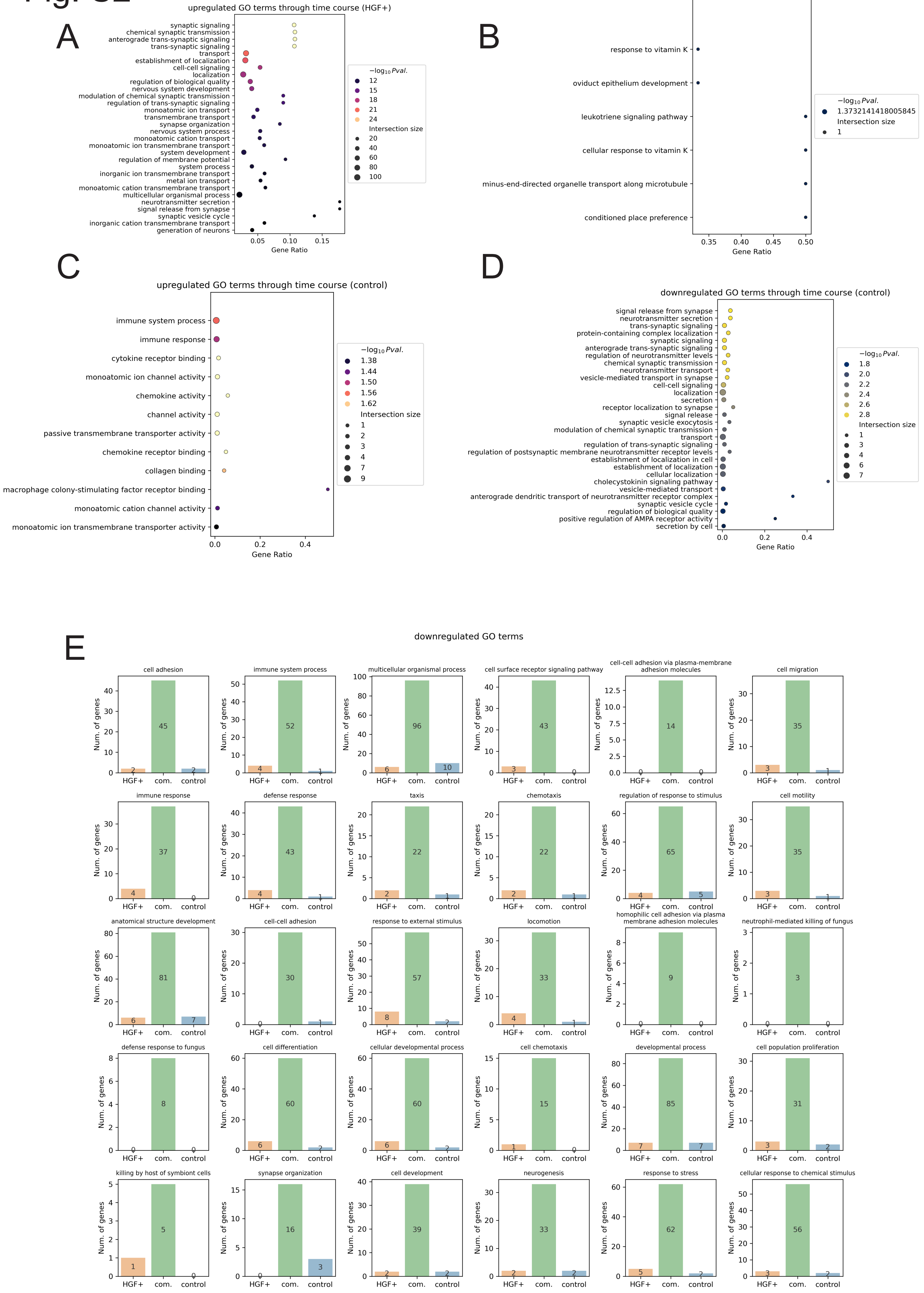
A
upregulated GO terms through time course (HGF+)
synaptic signaling
chemical synaptic transmission
anterograde trans-synaptic signaling
trans-synaptic signaling
transport
establishment of localization
cell-cell signaling
localization
regulation of biological quality
nervous system development
modulation of chemical synaptic transmission
regulation of trans-synaptic signaling
monoatomic ion transport
transmembrane transport
synapse organization
nervous system process
monoatomic cation transport
monoatomic ion transmembrane transport
system development
regulation of membrane potential
system process
inorganic ion transmembrane transport
metal ion transport
monoatomic cation transmembrane transport
multicellular organismal process
neurotransmitter secretion
signal release from synapse
synaptic vesicle cycle
inorganic cation transmembrane transport
generation of neurons
0.05
0.10
0.15
Gene Ratio
$-\log_{10} Pval.$
12
15
18
21
24
Intersection size
20
40
60
80
100
B
downregulated GO terms through time course (HGF+)
response to vitamin K
oviduct epithelium development
leukotriene signaling pathway
cellular response to vitamin K
minus-end-directed organelle transport along microtubule
conditioned place preference
0.35
0.40
0.45
0.50
Gene Ratio
$-\log_{10} Pval.$
1.3732141418005845
Intersection size
1
C
upregulated GO terms through time course (control)
immune system process
immune response
cytokine receptor binding
monoatomic ion channel activity
chemokine activity
channel activity
passive transmembrane transporter activity
chemokine receptor binding
collagen binding
macrophage colony-stimulating factor receptor binding
monoatomic cation channel activity
monoatomic ion transmembrane transporter activity
0.0
0.2
0.4
Gene Ratio
$-\log_{10} Pval.$
1.38
1.44
1.50
1.56
1.62
Intersection size
1
2
3
4
7
9
D
downregulated GO terms through time course (control)
signal release from synapse
neurotransmitter secretion
trans-synaptic signaling
protein-containing complex localization
synaptic signaling
anterograde trans-synaptic signaling
regulation of neurotransmitter levels
chemical synaptic transmission
neurotransmitter transport
vesicle-mediated transport in synapse
cell-cell signaling
localization
secretion
receptor localization to synapse
signal release
synaptic vesicle exocytosis
modulation of chemical synaptic transmission
transport
regulation of trans-synaptic signaling
regulation of postsynaptic membrane neurotransmitter receptor levels
establishment of localization in cell
establishment of localization
cellular localization
cholecystokinin signaling pathway
vesicle-mediated transport
anterograde dendritic transport of neurotransmitter receptor complex
synaptic vesicle cycle
regulation of biological quality
positive regulation of AMPA receptor activity
secretion by cell
0.0
0.2
0.4
Gene Ratio
$-\log_{10} Pval.$
1.8
2.0
2.2
2.4
2.6
2.8
Intersection size
1
3
4
6
7
E
downregulated GO terms
Num. of genes
HGF+
com.
control
cell adhesion: 2, 45, 2
immune system process: 4, 52, 1
multicellular organismal process: 6, 96, 10
cell surface receptor signaling pathway: 3, 43, 0
cell-cell adhesion via plasma-membrane adhesion molecules: 0, 14, 0
cell migration: 3, 35, 1
immune response: 4, 37, 0
defense response: 4, 43, 1
taxis: 2, 22, 1
chemotaxis: 2, 22, 1
regulation of response to stimulus: 4, 65, 5
cell motility: 3, 35, 1
anatomical structure development: 6, 81, 7
cell-cell adhesion: 0, 30, 1
response to external stimulus: 8, 57, 2
locomotion: 4, 33, 1
homophilic cell adhesion via plasma membrane adhesion molecules: 0, 9, 0
neutrophil-mediated killing of fungus: 0, 3, 0
defense response to fungus: 0, 8, 0
cell differentiation: 6, 60, 2
cellular developmental process: 6, 60, 2
cell chemotaxis: 1, 15, 0
developmental process: 7, 85, 7
cell population proliferation: 3, 31, 2
killing by host of symbiont cells: 1, 5, 0
synapse organization: 0, 16, 3
cell development: 2, 39, 2
neurogenesis: 2, 33, 2
response to stress: 5, 62, 2
cellular response to chemical stimulus: 3, 56, 2

Fig. S3

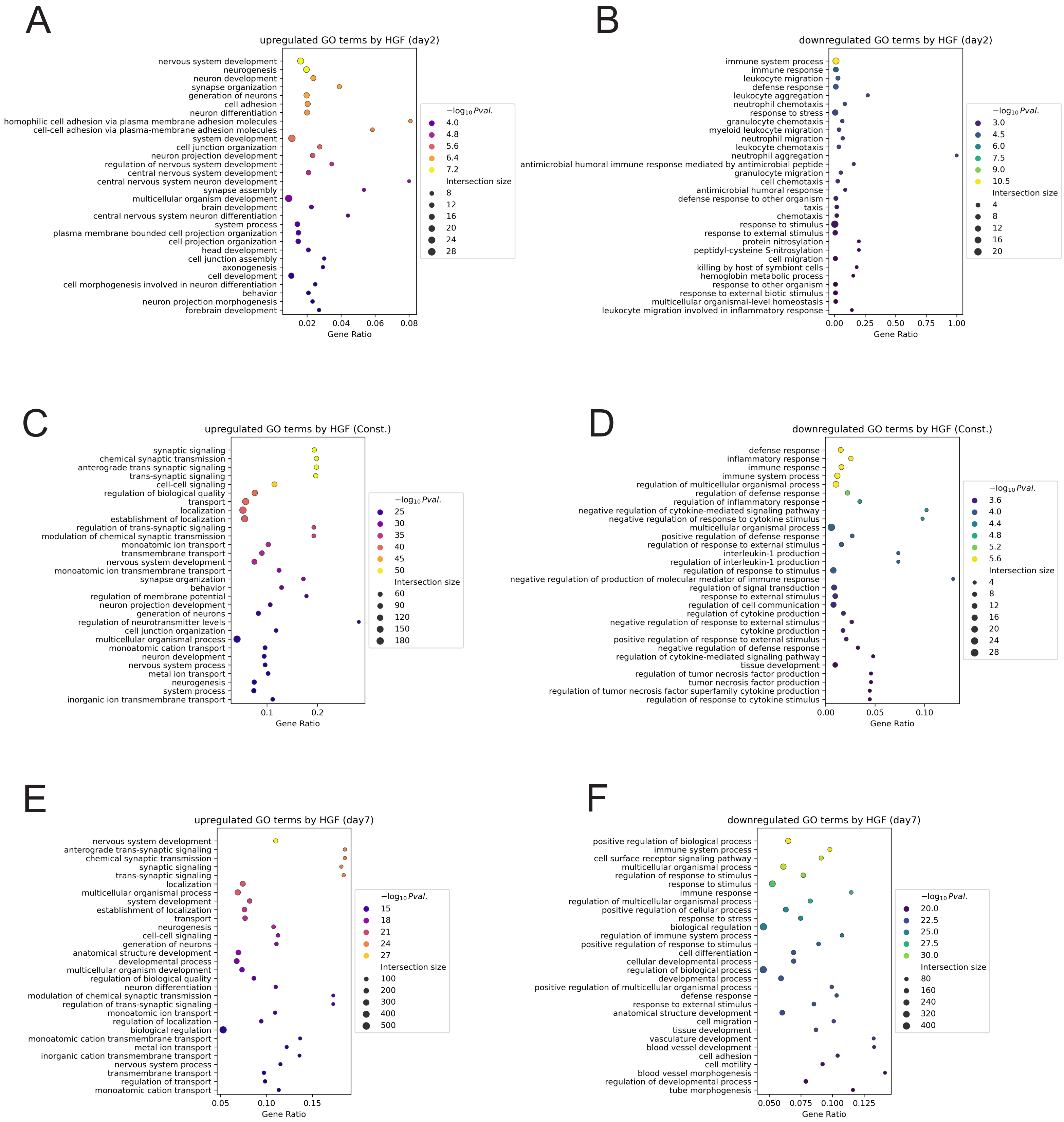
A
upregulated GO terms by HGF (day2)
nervous system development
neurogenesis
neuron development
synapse organization
generation of neurons
cell adhesion
neuron differentiation
homophilic cell adhesion via plasma membrane adhesion molecules
cell-cell adhesion via plasma-membrane adhesion molecules
system development
cell junction organization
neuron projection development
regulation of nervous system development
central nervous system development
central nervous system neuron development
synapse assembly
multicellular organism development
brain development
central nervous system neuron differentiation
system process
plasma membrane bounded cell projection organization
cell projection organization
head development
cell junction assembly
axonogenesis
cell development
cell morphogenesis involved in neuron differentiation
behavior
neuron projection morphogenesis
forebrain development
0.02
0.04
0.06
0.08
Gene Ratio
−log10 Pval.
4.0
4.8
5.6
6.4
7.2
Intersection size
8
12
16
20
24
28
B
downregulated GO terms by HGF (day2)
immune system process
immune response
leukocyte migration
defense response
leukocyte aggregation
neutrophil chemotaxis
response to stress
granulocyte chemotaxis
myeloid leukocyte migration
neutrophil migration
leukocyte chemotaxis
neutrophil aggregation
antimicrobial humoral immune response mediated by antimicrobial peptide
granulocyte migration
cell chemotaxis
antimicrobial humoral response
defense response to other organism
taxis
chemotaxis
response to stimulus
response to external stimulus
protein nitrosylation
peptidyl-cysteine S-nitrosylation
cell migration
killing by host of symbiont cells
hemoglobin metabolic process
response to other organism
response to external biotic stimulus
multicellular organismal-level homeostasis
leukocyte migration involved in inflammatory response
0.00
0.25
0.50
0.75
1.00
Gene Ratio
−log10 Pval.
3.0
4.5
6.0
7.5
9.0
10.5
Intersection size
4
8
12
16
20
C
upregulated GO terms by HGF (Const.)
synaptic signaling
chemical synaptic transmission
anterograde trans-synaptic signaling
trans-synaptic signaling
cell-cell signaling
regulation of biological quality
transport
localization
establishment of localization
regulation of trans-synaptic signaling
modulation of chemical synaptic transmission
monoatomic ion transport
transmembrane transport
nervous system development
monoatomic ion transmembrane transport
synapse organization
behavior
regulation of membrane potential
neuron projection development
generation of neurons
regulation of neurotransmitter levels
cell junction organization
multicellular organismal process
monoatomic cation transport
neuron development
nervous system process
metal ion transport
neurogenesis
system process
inorganic ion transmembrane transport
0.1
0.2
Gene Ratio
−log10 Pval.
25
30
35
40
45
50
Intersection size
60
90
120
150
180
D
downregulated GO terms by HGF (Const.)
defense response
inflammatory response
immune response
immune system process
regulation of multicellular organismal process
regulation of defense response
regulation of inflammatory response
negative regulation of cytokine-mediated signaling pathway
negative regulation of response to cytokine stimulus
multicellular organismal process
positive regulation of defense response
regulation of response to external stimulus
interleukin-1 production
regulation of interleukin-1 production
regulation of response to stimulus
negative regulation of production of molecular mediator of immune response
regulation of signal transduction
response to external stimulus
regulation of cell communication
regulation of cytokine production
negative regulation of response to external stimulus
cytokine production
positive regulation of response to external stimulus
negative regulation of defense response
regulation of cytokine-mediated signaling pathway
tissue development
regulation of tumor necrosis factor production
tumor necrosis factor production
regulation of tumor necrosis factor superfamily cytokine production
regulation of response to cytokine stimulus
0.00
0.05
0.10
Gene Ratio
−log10 Pval.
3.6
4.0
4.4
4.8
5.2
5.6
Intersection size
4
8
12
16
20
24
28
E
upregulated GO terms by HGF (day7)
nervous system development
anterograde trans-synaptic signaling
chemical synaptic transmission
synaptic signaling
trans-synaptic signaling
localization
multicellular organismal process
system development
establishment of localization
transport
neurogenesis
cell-cell signaling
generation of neurons
anatomical structure development
developmental process
multicellular organism development
regulation of biological quality
neuron differentiation
modulation of chemical synaptic transmission
regulation of trans-synaptic signaling
monoatomic ion transport
regulation of localization
biological regulation
monoatomic cation transmembrane transport
metal ion transport
inorganic cation transmembrane transport
nervous system process
transmembrane transport
regulation of transport
monoatomic cation transport
0.05
0.10
0.15
Gene Ratio
−log10 Pval.
15
18
21
24
27
Intersection size
100
200
300
400
500
F
downregulated GO terms by HGF (day7)
positive regulation of biological process
immune system process
cell surface receptor signaling pathway
multicellular organismal process
regulation of response to stimulus
response to stimulus
immune response
regulation of multicellular organismal process
positive regulation of cellular process
response to stress
biological regulation
regulation of immune system process
positive regulation of response to stimulus
cell differentiation
cellular developmental process
regulation of biological process
developmental process
positive regulation of multicellular organismal process
defense response
response to external stimulus
anatomical structure development
cell migration
tissue development
vasculature development
blood vessel development
cell adhesion
cell motility
blood vessel morphogenesis
regulation of developmental process
tube morphogenesis
0.050
0.075
0.100
0.125
Gene Ratio
−log10 Pval.
20.0
22.5
25.0
27.5
30.0
Intersection size
80
160
240
320
400
